# The Use of Echocardiography in the Critically Ill; The Role of FADE (Fast Assessment Diagnostic Echocardiography) Training

**DOI:** 10.2174/157340311798220449

**Published:** 2011-08

**Authors:** Susan Marum, Susanna Price

**Affiliations:** UCII, Instituto Português de Oncologia Francisco Gentil, Lisbon

**Keywords:** Echocardiography, FADE, visualisation, haemodynamic status, intrapulmonary.

## Abstract

Echocardiography (echo) is a powerful technique that permits direct visualization and assessment of all the cardiac structures and assessment of the patients’ haemodynamic status at the bedside. Echo allows detection of valvular disease, evaluation of ventricular function and the pericardium, detection of intracardiac/intrapulmonary shunts, and can be used to calculate flows and relative pressures between the cardiac chambers. This rapid point-of-care haemodynamic evaluation provides information to guide therapeutic interventions, including volume resuscitation, instigation of vasoactive therapy and/or referral for specialist cardiac/surgical intervention. Although there is abundant evidence in the cardiology literature regarding the use of echo, data in the critical care arena is less well defined, but emerging. The use of echo by intensive care doctors is likely to become routine, and therefore training for intensivists in this technique needs to be developed and supported. The Portuguese Working Group on Echocardiography has developed a skill-based program, FADE (**F**ocused **A**ssessment **D**iagnostic **E**chocardiography) in order to train clinicians in the use of bedside ultrasound as a diagnostic and monitoring tool for the critically ill.

## INTRODUCTION

Although regarded classically as the domain of cardiologists, in recent years the use of echo has extended to other specialties, in particular anaesthesia. Here, the requirement to perform a transoesophageal echo (TOE) in order to monitor cardiac surgery provided the impetus to develop specific training for cardiothoracic anaesthetists in the technique. Currently accreditation in TOE exists from a number of bodies, and in certain countries this accreditation is mandatory to obtain a consultant post. The importance of echocardiography in the intensive care unit (ICU) has been recognized by a number of scientific societies (ASE, BSE, ILCOR, ESC, WINFOCUS), and the advantages are summarized in Table **1**. Despite the potential uses, obtaining training in echocardiography remains a challenge for most intensivists, and the first accreditation in intensive care echocardiography was only piloted in 2010 by the British Society of Echocardiography (BSE). This accreditation recognises that the questions faced by intensive care physicians are different from those in the out-patient echocardiography department, even for relatively common indications, such as the assessment of valvular pathology and ventricular function. Further, the technical challenges presented by intensive care unit (ICU) imaging are substantial, including sub-optimal lighting conditions, challenges in patient positioning, patient weight gain (oedema and/or surgical emphysema), chest drains, abdominal/chest dressings, positive pressure ventilation and rapidly changing haemodynamic support and ventilatory settings.

Table **2** summarises the most frequent questions to which an echocardiogram can give an answer in the ICU. The information is based on the possibilities of image acquisition in the ICU setting, where as a rule, examinations are performed in non-ideal conditions and non-ideal patients as already mentioned. In a recent work published by our group we observed that cardiac chamber dimensions could be obtained in 97.8% of the patients, cardiac output in 86.7%, left ventricular shortening fraction in 95.2%, and inferior vena cava evaluation in 65%. Conditions affecting echocardiographic performance are characterized: weight gain (excessive fluids and generalized oedema), presence of chest tubes, and presence of abdominal bandages (affecting subcostal views). This data was in general confirmed by other authors [1].

**Fig. (1):**
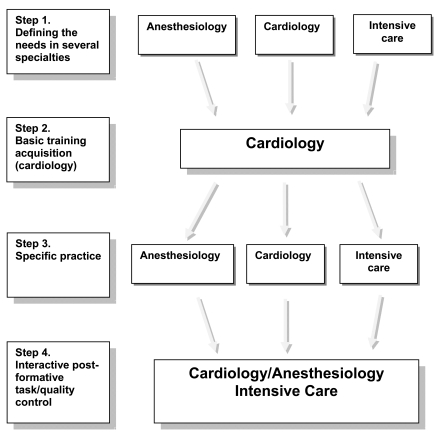
An integrative model of interaction between several specialties during echocardiography training acquisition.

Finally, there is no agreed minimum dataset for all intensive care echocardiography studies, as the groups of patients admitted relating to the specific ICU (i.e. trauma/general medical/ cardiac surgical) demand particular expertise. Thus, although many recommendations support the principle of training in echocardiography for ICU physicians, the preferential approach (TTE vs. TOE) and the minimum dataset are by definition not uniform. Further, there are marked national differences in training and accreditation programs – even for cardiologists (Fig. **1**). 

In Portugal there is no National accreditation in echocardiography, with the only option for training being to undertake a fellowship/training programme in another country. In this paper we outline a Portuguese training programme which follows the classification recommended by the ASE^[2,3]^, WINFOCUS and the French Echo group^[4,5]^ (Tables **3** & **4**)). These delineate three levels of training/competence: Level 1 –basic, Level 2 – advanced, Level 3 – highly skilled. In terms of defining content, the global initiative Winfocus has gathered specialists from different countries and published a comprehensive description of the syllabus that corresponds to each step of training in echo for the intensivist^[6]^. The widespread acceptance of such formative programmes is the key for critical care echo. Further, extension of ultrasonographic evaluation to the lungs is considered to be an important skill for the ICU clinician.

## LUNG ULTRASOUND

The validity of lung ultrasound beyond assessment of pleural fluid is not yet widely recognised, however, there is huge potential for this imaging modality in the critically ill. Indeed, the reported sensitivity and specificity of lung US in the diagnosis of pleural fluid and pneumothorax is significantly higher than that of plain chest radiography, and approaches that of CT scanning. In addition to its diagnostic value, thoracic US can be used to guide drainage of pleural collections and pneumothorax.

The stages involved in lung US of the critically ill have been well described by Daniel Lichtenstein^[7]^ and its potential use is outlined in Table **5**. 

## THE FADE PROGRAMME

FADE is a formative programme dedicated to training to Level 1 competency in echocardiography and chest ultrasound for the intensive care clinician. Over a two day course, a combination of blended learning techniques are employed, including; hands-on training on live models, performance of studies on the ICU, theoretical lectures, and clinical case discussions of recorded examinations. The first day introduces the principles of the basic echocardiography examination in normal models, and the second focuses on the use of echocardiography in the management of the haemodynamically unstable patient. Following attendance at the FADE course, trainees undertake additional mentored study in order to demonstrate competency in acquisition and interpretation of TTE views in a critical/emergency setting. This enables them to identify major causes of hypotension, shock, respiratory failure and recognise when referral for a second opinion is indicated. The program is outlined in detail in Table **6**. 

## CONCLUSION

Although evidence that ICU echo is valuable is emerging, training of intensive care clinicians in this technique still remains challenging. Training and accreditation programmes aiming to deliver Level 1 competencies tailored to national requirements and sensitivities are emerging in many countries (i.e. FATE: Denmark, FEEL-United Kingdom, FEEL: Germany, French ICU echo: France). In Portugal the FADE programme has been developed in order to address this challenge, providing a two-day training program as an introduction and opening the path to Level 1 of the recognised competencies in critical care ultrasound.

## Figures and Tables

**Table 1.** The Advantages and Disadvantages in Performing Echocardiography in the ICU

Advantages	Disadvantages
The information is acquired in real-time No health care practitioners are needed other than the performing physician The information is obtained before the invasive monitoring	Does not provide potential for continuous monitoring In several patients it is not possible to acquire all the classic echocardiographic views

**Table 2.** Information Frequently Requested from Echocardiography in the General Intensive care

Information	Echocardiographic View

LV systolic function	Paraesternal long axis and short axis view, 2, 3 and 4-chamber view
Cardiac output	4-chamber view
Right heart assessment	Paraesternal long axis and short axis view, 4-chamber view
Pericardial disease	Paraesternal long axis and short axis view, 4-chamber view, subcostal view
Valvular disease	Paraesternal long axis and short axis view, 4-chamber view
Volume status and responsiveness	4-chamber view, inferior vena cava

**Table 3.** The Formative Program Based on the Recommendations of the American Echocardiography Society

Level 1. Basic experience, includes the performance of 150 transthoracic examinations, and a minimum period of 3 months.
Level 2. Experience toward a autonomous echocardiography performance. Requires na additional 150 transthoracic examinations in a 3 month-period.
Level 3. Advanced performance, requires na additional performance of 450 echocardiogramms in a 6 month period.

**Table 4.** The training in Echocardiography for the French Society of Intensive Care

Level 1. Introduction to the technique, during a 3-month period and performance of 120 examaminations
Level 2. Autonomous performance of echocardiograms, during a 3-month period and 120 examinations. Introduction of specific training for the Intensive Care physician
Level 2. Training in transesophageal echocardiography during a 3-month period and 120 examinations. Introduction of specific training for the Intensive Care physician
Level 3. Long duration training for Laboratory directors; no defined time period or number of examinations

**Table 5.** Possibilities of Thoracic Ultrasound

Diagnosis of pleural effusion Quantification of pleural effusion Characterization of pleural effusion Identification of pleural masses Identification of parenchymal disease (infection or masses) Identification of pulmonary oedema

**Table 6.** FADE PROGRAMME

Day	Theoretical Training	Practical Training

Day 1	Physics of ultrasound	Hands-on training
		PLAX
		PSAX
		A4Ch
		SC

	US anatomy of the heart & lungs: PLAX/PSAX, A4Ch, SC, IVC, thoracic	

	Basic assessment:	
	LV function (global & regional)	
	RV function	
	Valves	
	Pericardium	
	IVC	
	CO	

	Pitfalls of assessment in the critically ill	

	Lung US:	
	Pleural effusion	
	Pneumothorax	

Day 2	LV in the ICU; sepsis & related syndromes	ICU hands-on training;
		Supervised studies in the ICU including measurement of CO, combined with clinical discussion

	IPPV and the heart	Interpretation of recorded clinical cases

	IVC and derived parameters	

	Echo in shock states	

	Dynamic indices of volaemia	

	Weaning from mechanical ventilation	

Assessment	Interpretation of snapshot images within clinical context	Obtaining echocardiographic views in live models
